# Electrotactile BCI for Top-Down Somatosensory Training: Clinical Feasibility Trial of Online BCI Control in Subacute Stroke Patients

**DOI:** 10.3390/bios14080368

**Published:** 2024-07-28

**Authors:** Andrej M. Savić, Marija Novičić, Vera Miler-Jerković, Olivera Djordjević, Ljubica Konstantinović

**Affiliations:** 1 University of Belgrade – School of Electrical Engineering, 11000 Belgrade, Serbia; 2 Innovation Center of the School of Electrical Engineering, University of Belgrade, 11000 Belgrade, Serbia; 3 University of Belgrade – Faculty of Medicine, 11000 Belgrade, Serbia; 4 Clinic for Rehabilitation “Dr Miroslav Zotović”, 11000 Belgrade, Serbia

**Keywords:** brain–computer interface (BCI), clinical study, electrical stimulation, tactile attention, somatosensory training, somatosensory event-related potentials (sERPs), subacute stroke

## Abstract

This study investigates the feasibility of a novel brain–computer interface (BCI) device designed for sensory training following stroke. The BCI system administers electrotactile stimuli to the user’s forearm, mirroring classical sensory training interventions. Concurrently, selective attention tasks are employed to modulate electrophysiological brain responses (somatosensory event-related potentials—sERPs), reflecting cortical excitability in related sensorimotor areas. The BCI identifies attention-induced changes in the brain’s reactions to stimulation in an online manner. The study protocol assesses the feasibility of online binary classification of selective attention focus in ten subacute stroke patients. Each experimental session includes a BCI training phase for data collection and classifier training, followed by a BCI test phase to evaluate online classification of selective tactile attention based on sERP. During online classification tests, patients complete 20 repetitions of selective attention tasks with feedback on attention focus recognition. Using a single electroencephalographic channel, attention classification accuracy ranges from 70% to 100% across all patients. The significance of this novel BCI paradigm lies in its ability to quantitatively measure selective tactile attention resources throughout the therapy session, introducing a top-down approach to classical sensory training interventions based on repeated neuromuscular electrical stimulation.

## 1. Introduction

Stroke is the second leading cause of disability and death globally [1]. Among stroke survivors, sensory deficits, including touch discrimination issues, are prevalent, affecting 50%–85% of individuals [2]. Somatosensory impairment has a significant negative impact on rehabilitation outcomes and post-stroke functionality in general [3,4]. Various somatosensory training interventions are designed to retrain sensory function [5]. These interventions involve diverse methods, such as touching textured objects, massage, pressure application and different sensory discrimination tasks [6]. The current technical solutions for providing the necessary afferent stimulation involve rehabilitation robotics, vibration devices or transcutaneous electrical stimulation [7].

The multitude of previous studies have explored interventions utilizing somatosensory electrical stimulation below the motor threshold, delivered to the paretic limbs of stroke patients. These interventions aim to treat sensory deficits or enhance the benefits of other neurorehabilitation treatments by facilitating motor learning through the activation of the sensorimotor feedback loop [7]. However, these interventions are primarily “bottom-up”, focusing on distal limb stimulation to induce reorganization in neural circuits and facilitate the recovery process [8]. Recent trends in neurorehabilitation propose the introduction of “top-down” interventions to more effectively induce, direct and monitor brain plasticity mechanisms during the rehabilitation process [8]. There is a growing body of evidence highlighting the importance of voluntary top-down attention in stimulation-induced somatosensory cortex plasticity and gating perceptual learning [9,10].

Brain–computer interfaces (BCIs) offer the potential of introducing top-down approach in neurorehabilitation by employing neurofeedback practices [11]. In current BCIs for stroke rehabilitation, afferent stimulation is provided as a feedback modality, triggered by the executed mental or motor task detected and/or classified by the BCI [12,13]. This approach has been shown to facilitate activity-dependent brain plasticity in the sensorimotor cortex [14]. However, no previous studies have proposed or tested BCI-based somatosensory training protocols after stroke that exclusively target sensory cortex activation through voluntary top-down attention mechanisms. We introduced a novel BCI-based somatosensory training intervention that integrates a top-down approach into classical sensory training based on repeated neuromuscular electrical stimulation. Our BCI enables the online classification of voluntary selective spatial attention toward one of the two stimulation locations over the forearm muscles of the paretic upper limb (UL). This occurs during a sensory stimulation protocol that involves delivering repetitive pulsed electrotactile stimuli sequentially to targeted paretic UL muscles. Voluntary selective attention is detected and quantified from users’ electroencephalographic (EEG) data. The primary goal of this BCI is to improve classical somatosensory interventions (i.e., discrimination tasks employing repetitive pulsed electrical stimulation) by engaging the user’s voluntary attention resources and directing them toward the task while quantifying the user’s attention resources from the EEG. Hence, the BCI provides online feedback to the user on the success of recognizing tactile attention tasks. More specifically, it calculates online objective EEG-based measures of the user’s voluntary selective attention and provides feedback to the user and/or therapist, with an aim to verify that the user is executing the mental task in an expected manner. Moreover, this approach enables the user to train and correct the mental strategy if necessary. This setup allows a unique window to track online the direct measures of the user’s top-down attentional engagement during the therapeutic intervention, which was proven to have a direct impact on the therapy outcome.

The overall aim of this paper is to describe the novel BCI-based sensory training method, its validation and clinical implementation pipeline, along with the novelties it introduces in the fields of somatosensory training interventions and BCI systems in general. To the best of our knowledge, this work represents the first attempt in designing a BCI system specifically for improving somatosensory training interventions after stroke. Furthermore, our goal is to provide initial evidence of the clinical feasibility and deployment potential of such intervention. In our study, we introduce a novel concept with a potential to improve the traditional sensory rehabilitation after stroke by integrating top-down approaches through electrotactile BCI. This is the first reactive, tactile BCI system employing electrical stimuli for control, tested in stroke patients with sensory deficits. This paper presents empirical novelties by presenting and analyzing the patterns of somatosensory event-related potentials (sERPs), recorded for the first time from subacute stroke patients during selective spatial tactile attention tasks, offering insights into the underlying neural responses associated with our novel BCI paradigm. Additionally, our study assesses the online classification performance, offering the first evidence of the feasibility of this concept in the target population of subacute stroke survivors. Finally, this study aimed to determine whether the patients’ functional status had significant effect on BCI performance, since it is known that the presence of stroke-related lesions may modify the control signals and affect the BCI performance [15].

### Theoretical Background on Neural Responses to Somatosensory Stimulation

Voluntary attention is a key mechanism for selecting relevant sensory information for detailed and effective processing and actively suppressing distracting, irrelevant sensory information [16]. Studies have demonstrated that voluntary selective attention toward tactile stimuli modifies electrophysiological brain responses, hemodynamic neural activity and cortical excitability in related sensorimotor areas [17,18,19,20]. Moreover, multiple pieces of evidence suggest that the potential for inducing plasticity in the somatosensory brain area increases when selective spatial attention is directed toward stimuli targeting the contingent limb [21]. However, all those studies only dealt with offline neural data analysis while neither proposing nor exploring the potential of online measurement and quantification of tactile attention resources during a sensory intervention to make it more efficient via neurofeedback.

Somatosensory stimulation can elicit various responses measured by EEG, including transient somatosensory evoked potentials (SEPs) and steady-state somatosensory evoked potentials (SSSEP), which can be used for online quantification of tactile attention [22]. SEPs, routinely used in clinical practice, result from electrical stimulation of peripheral nerves and consist of a series of waves reflecting sequential activation of neural structures along somatosensory pathways [23]. SSSEP signals have been previously utilized for BCI control in various studies [22]. In contrast to SSSEP, when the stimulation rate is set to evoke transient SEPs, and when such stimulation is coupled with a cognitive task (such as a selective attention task), the responses are termed somatosensory event-related potentials (sERPs). The induction and recording methods for sERPs may be identical to scalp measurements of classical SEPs [24].

BCIs can be categorized into three main types based on the nature of their interaction and control mechanisms: active, reactive and passive [25]. Active BCIs rely on the user’s intentional and self-generated brain activity. Reactive BCIs measure the changes in a user’s brain activity in response to external stimuli that the user needs to attend to, while passive BCIs monitor the user’s brain activity without the need for intentional control. Our BCI system relies on sERP responses to external electrotactile stimuli and can therefore be classified as a reactive system. More specifically, our reactive BCI system measures the immediate effects of the selective tactile attention task through the online extraction of sERP features from EEG signals.

## 2. Methods

The study was approved by the Ethics Committee of the Clinic for Rehabilitation “Dr. Miroslav Zotović” affiliated with the Faculty of Medicine, University of Belgrade, under the ethics approval number 03-1514/1 (date: 3 July 2020). It was registered at ClinicalTrials.gov prior to participant enrollment (NCT number: NCT05152108; official title: “Feasibility Clinical Study of a Novel Hybrid Brain–Computer Interface for Control of Sensory-Motor Coupling in Post-Stroke Rehabilitation”). The study type is defined as interventional (BCI clinical feasibility trial), with the primary outcome measure being the accuracy of BCI operation. All participants signed the informed consent form, while all research procedures were performed in accordance with the Declaration of Helsinki.

### 2.1. Patient Recruitment and Assessment

All subjects were admitted to the inpatient ward for neurorehabilitation at the Clinic for Rehabilitation “Dr. Miroslav Zotović”, Belgrade, Serbia. The inclusion criteria for this study were as follows: age over 18 years at the time of enrollment; cerebrovascular insult (CVI) verified by computerized tomography or magnetic resonance imaging; CVI occurring no less than 2 weeks and more than 6 months prior to study enrollment (patients in the subacute phase of recovery); medically stable patients, as determined by medical history and documented neurological examination; ability to understand instructions and communicate with the research team; ability to adhere to the study protocol. The exclusion criteria included any neurological condition (beyond stroke) or physical condition that additionally affected the function of the impaired arm; a substantial cardiopulmonary or metabolic disorder or other major medical complication; history of seizures; moderate-to-severe hemispatial neglect or anosognosia involving the affected arm; severe spasticity, defined as an Ashworth scale score of 4 in the affected arm; nursing a child, pregnancy.

A clinical assessment of the patients’ status was conducted the day before the tests involving the BCI system. The following clinical measures were obtained: Fugl-Meyer motor score for the upper extremity (FM-m), Fugl-Meyer sensory score for the upper extremity (FM-s), action research arm test (ARAT), Barthel index (BI), the modified Ashworth scale (AS), two-point discrimination test (2-PD) and stereognosis examination.

The Fugl-Meyer motor score for the upper extremity (FM-m) assesses the degree of synergistic movements in the paretic upper limb. Individual items pertaining to the shoulder/elbow and hand segments are scored on a 3-point ordinal scale and summed for a maximum possible score of 66. The Fugl-Meyer motor score for the upper extremity is reported as a total score. A higher score is considered to represent better performance of the subject.

The Fugl-Meyer sensory score for the upper extremity (FM-s) assesses the degree of sensory impairment in the upper arm. Individual items pertaining to the light touch sensation of the upper arm/forearm and palmar surface of the hand, as well as the position of the shoulder/elbow/wrist/thumb, are scored on a 3-point ordinal scale and summed for a maximum possible score of 12. A higher score is considered to represent lower sensory deficit in the subject [26].

The action research arm test (ARAT) measures specific changes in arm function in individuals who sustained cerebral damage resulting in arm weakness. The ARAT consists of 19 items grouped into four subscales: grasp, grip, pinch and gross movements. The performance of the subjects in each subscale is scored on a four-level ordinal scale. The sums of all subscales are added to compute the total score, which ranges from 0 to 57. A higher score is considered a better outcome [27].

The Barthel index (BI) measures the degree of assistance required by a person for ten mobility and self-care activities of daily living [28]. The ten items are scored with a number of points. A sum score is calculated by summing the points awarded to each functional activity. Higher scores reflect higher independence of the patient in completing the measured activities of daily living. The maximum possible score is 100.

The Ashworth scale (AS) is a rating scale to measure abnormality in muscle tone or resistance to passive movements. It is a 6-point ordinal scale [29]. A higher score indicates a higher level of muscle tone.

In the two-point discrimination test, the examiner uses a paper clip and touches two points on the fingertips of the paretic side. The minimum distance the patient can distinguish between two stimuli on the fingertip is normally <6 mm, while the smallest distance between two points that still results in the perception of two distinct stimuli is recorded as the patient’s two-point threshold [30]. The hand being tested is immobile, placed on a hard surface and not seen by the subject.

Stereognosis, or the ability to identify a three-dimensional object by tactile manipulation of that object in the absence of other sensory stimuli, is tested by a tactile object recognition test. It involves placing a series of common objects (coin, key, marble, pen, comb, paper clip) in the patients’ hands and asking them to identify the object with their eyes closed. If they are unable to identify the object despite having intact peripheral sensory function, they are considered to have astereognosis [31,32].

Table 1 includes the demographic and clinical assessment data of the 10 subacute stroke patients recruited for the clinical feasibility study.

### 2.2. Experimental Setup

The participants were comfortably seated in a chair with a computer screen approximately 1 m away, positioned on a desk in front of them. The tests were conducted by a team comprising experienced clinicians specialized in physical medicine and rehabilitation, along with biomedical engineers experienced in operating the novel BCI system. The BCI device was operated through a custom graphical user interface developed in MATLAB R2020a (MathWorks Inc., Natick, MA, USA).

The EEG signals were recorded using the g.USBamp electrophysiological signal amplifier and active electrodes (g.GAMMAcap2) with pre-amplification (g.tec GmbH, Schiedlberg, Austria). Six 10–20 EEG recording sites were utilized based on the patient’s body side impairment. For right-side impairment, the EEG sites were FP1, C3, Cz, C4, CP5 and P3, while for left-side impairment, the channel locations were FP1, C3, Cz, C4, CP4 and P4, covering the ipsilesional sensorimotor area (i.e., cortical area contralateral to the affected upper limb) with additional EEG channels. This was performed because the main sources of EEG activity in response to somatosensory stimuli are expected in the somatosensory cortical area contralateral to the stimulated upper limb, which was selected in this study based on the impairment side. The reference electrode was placed on the left earlobe and the ground at AFz. FP1 location was used to register ocular artifacts. The signals were acquired with a sampling rate of 1200 Hz. Subjects were instructed to limit body movements during the experiment, avoid excessive and systematic blinking and fix their gaze at the fixation cross displayed on the computer screen to minimize ocular movements.

**Figure 1:**
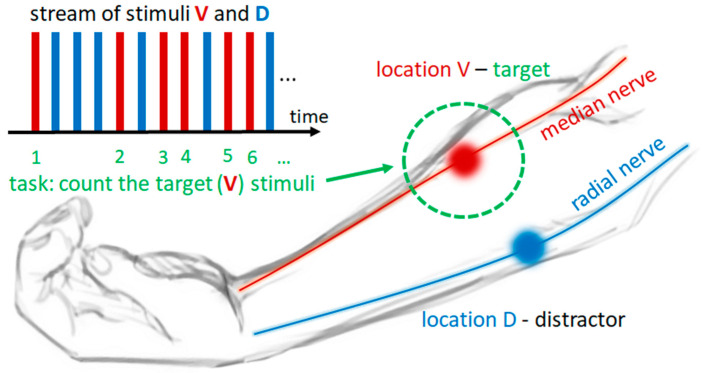
Schematics of the experimental task and stimulation electrodes’ layout. Electrical stimulation hotspots, i.e., the locations of stimulation electrodes on the dorsal and volar surfaces of the forearm, are depicted with blue and red circles, respectively. Stimulation location D (blue) is located over the radial nerve, and stimulation location V (red) is located over the median nerve at the forearm. In this example, location V is the target location (marked by dotted green circle), and location D is the distractor location. Electrotactile stimuli are represented by blue and red rectangles, where blue rectangles depict stimuli delivered to location D, and red rectangles depict stimuli delivered to location V, while their stream represents the temporal sequence (from left to right) of stimuli delivery to each location in a sequential manner. The subject’s task—counting the stimuli delivered to target location—is presented by numbering each red rectangle in a sequence, while the blue rectangles are ignored.

Electrical stimulation was delivered using two channels of the MOTIMOVE electrical stimulator (3F—Fit Fabricando Faber, Belgrade, Serbia). The electrical stimuli consisted of single, constant current, compensated biphasic pulses with exponential discharge. The pulse duration in the active phase was 0.25 ms. Three electrical stimulation electrodes (Axelgaard Manufacturing Co., Ltd., Lystrup, Denmark) were positioned on the affected forearm of the patient. Two active electrodes (round shape, 1 cm diameter) were placed on the dorsal and volar surfaces of the affected forearm, while a common indifferent electrode (round shape, 2.5 cm diameter) was placed on the volar aspect of the right wrist. The dorsal active electrode was positioned over the extensor carpi radialis muscle (location D), and the volar active electrode was placed over the flexor carpi radialis longus (location V). Location D was identified at the proximal 20% of the line length from the lateral humeral epicondyle to the radial styloid with the forearm in pronation. Location V was identified at the proximal 33% of the distance connecting the medial epicondyle and the base of the second metacarpal bone with the forearm in supination. The motor threshold for both stimulation sites was determined by gradually increasing the pulse amplitude starting from 5 mA, in 1 mA steps, and inspecting visible muscle activations for D and V. If necessary, stimulation electrodes were slightly repositioned around the predefined hotspot until selective activations of flexor carpi radialis longus and extensor carpi radialis were successfully achieved. Subsequently, the stimulation amplitudes were slightly reduced below the motor threshold and balanced between the two sites to ensure a similar subjective feeling of electrical stimulation intensity. The stimulation was maintained below the pain threshold and visible motor contraction. Patients actively participated in the electrical stimulation intensity balancing process. Single pulses were consecutively delivered at both D and V, and the patients were asked to compare the sensation intensity. If the sensation was stronger at one site, the pulse amplitude was decreased. This process was repeated until balanced sensations were achieved, and the final pulse amplitudes for both sites were noted by the experimenter. The EEG and electrical stimulation electrodes’ positioning and relevant parameters’ setup were previously explained and validated in studies conducted with healthy participants [33,34]. Figure 1 shows the schematics of the stimulation electrodes’ layout and visual depiction of one selective spatial attention task performed by the subject, as explained in the following section.

### 2.3. Experimental Protocol

To facilitate further explanations, we introduce the following terms that will be used in the following sections to explain the experimental protocol and EEG processing and feature extraction procedures:

- An “epoch” refers to an EEG response to a single electrical stimulus delivered either at location V or D.
- The “sERP” describes an average over a specific number of epochs delivered at the same location. Consequently, “sERP” can originate from stimulated location V or D.
- A “block” refers to a sustained attention task at a single stimulated location, during which a set of electrical stimuli is delivered to both locations sequentially in a randomized order. During each block, 30 electrical stimuli are delivered (15 per location), and 15 epochs per location are generated and later utilized for tactile attention classifier training.
- A “trial” refers to a task/procedure structured similarly to a block, with the difference that each trial is classified online, and the result of the classification is presented to the subject.

**Figure 2:**
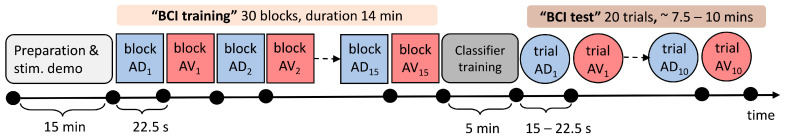
Timeline of the experimental protocol. The colored square shapes represent the experimental blocks of the BCI training phase, while the colored circular shapes represent the classification trials of the “BCI test phase”. In each block or trial, electrical stimuli were delivered in a randomized order to locations D and V. Within each block/trial, subjects were instructed to attend to stimuli delivered to D or V, which are color-coded—blue for attending to location D (AD) and red for attending to location V (AV)—while the block/trial number is given in the subscript. The durations of experimental protocol elements are given on the time axis below separate shapes. The number of electrical stimuli delivered in each block is 30, 15 per location D and V; therefore, the duration of each block is fixed to 22.5 s, while the total duration of the BCI training phase, including 5 s pauses between the blocks, is around 14 min. The number of electrical stimuli delivered in each trial varied between 20 and 30 depending on the number of rejected epochs in order to obtain 10 noise-free epochs per location for average sERP. The trial duration varied between 15 and 22.5 s, while the total duration of the BCI test phase, including 5 s pauses between the trials, varied between 7.5 and 10 min. The gray rectangular shapes denote the patient preparation phase, including EEG and ES system placement and setup and task explanation/demonstration (light gray), as well as classifier training between BCI training and test phases in which the patient was resting. The total duration of the experiment was around 45 min.

The timeline of the experimental protocol is graphically presented in Figure 2. We emphasize that the protocol presented here reflects the realistic clinical application pipeline for a new sensory intervention. The first phase, termed the “BCI training phase”, was dedicated to training the BCI classification of two selective spatial tactile attention tasks solely from EEG signals. This phase comprised 30 blocks represented by square shapes in Figure 2, with 15 blocks per selective attention task. In each block, 30 single electrical pulse stimuli were sequentially delivered in a randomized order to locations D and V with a 700 ms inter-stimulus interval. During each block, the patient’s task was to perform a sustained spatial tactile attention task, selectively attending to the stimuli delivered to a single target location D or V by silently counting the stimuli while ignoring those arriving at the other location (distractor). The tasks alternated between attending to D location stimuli (AD) or attending to V location stimuli (AV). Figure 1 shows an example of a stream of electrotactile stimuli and of one selective attention task, where location V was the target and location D the distractor.

Before the start of each block, the experimenter announced the task (AD or AV), and instructions were given to the patient to silently count the stimuli delivered to the current target location. Each block began with a countdown from 3 to 0 on the computer screen, followed by the appearance of the fixation cross, indicating the start of the stimulation sequence. Target and distractor locations alternated between consecutive blocks, with the starting target location randomized among patients. In short pauses between the blocks, subjects reported the counted number of stimuli and confirmed the previously attended location by pointing to it with the finger of the unaffected hand. This process ensured that the subject counted the expected number of stimuli and followed the instructions correctly.

At least one “demonstration block” for each target location was conducted with each subject before the experiment began. The purpose was to demonstrate the stimulation sequence, explain the task and verify the correct understanding of the protocol. After completing the BCI training phase, the experimenter initiated classifier training via the graphical user interface (GUI) of the BCI software application. The subject rested for 5 min while the recorded EEG training data were processed and classifiers trained before starting the second phase, the “BCI test phase”, of the experiment.

In this phase, the patients performed 20 attempts (trials) of the same selective spatial attention task (10 trials per target location D or V), while the stimuli were delivered in the same manner as in the training phase (depicted by circular shapes in Figure 2). Each trial lasted until the features for classification were acquired, calculated from 10 noise-free sERP averages for each location. Therefore, the system detected and removed noisy epochs in an online manner and delivered stimuli to both locations until 10 valid epochs were collected per location, enabling their averaging to obtain the sERP. The online BCI performance was tested on 20 classifications (trials) of selective spatial attention by the previously trained BCI system. Each online classification trial, i.e., an attempt of online classification of a spatial attention task, was followed by displaying a message on the computer screen about the classification result: correct or incorrect target location recognized from the EEG. This provided feedback to the subject and experimenter, followed by the announcement of the next target location, with a countdown from 3 to 0 indicating the start of the next online classification trial.

### 2.4. EEG Signal Processing and Feature Extraction

After the conclusion of the “BCI training phase”, while the patient was resting, the EEG data were processed to obtain sERP responses elicited by electrical stimuli. The training phase involved two distinct attention tasks—sustained attention to stimuli delivered at the dorsal location (target D, attended D—AD) and sustained attention at the volar location (target V, attended V—AV)—and during each attention task, stimuli were delivered to both locations. This resulted in four sERP responses associated with the following conditions:

- D location is stimulated, D location is the target: ADSD (attended D, stimulated D);
- V location is stimulated, D location is the target: ADSV (attended D, stimulated V);
- D location is stimulated, V location is the target: AVSD (attended V, stimulated D);
- V location is stimulated, V location is the target: AVSV (attended V, stimulated V).

The recorded EEG data were initially bandpass-filtered using a fourth-order Butterworth filter with cut-off frequencies of 0.1 and 25 Hz. Subsequently, the data were segmented into 700 ms epochs, including a 100 ms pre-stimulus baseline and a 600 ms post-stimulus window. All epochs were baseline-corrected by subtracting the mean value of the 100 ms baseline from the post-stimulus window values. Epochs containing amplitude drifts were rejected by applying a threshold of ±50 µV on five somatosensory EEG channels. Moreover, ocular artifacts were identified by applying a threshold of ±80 µV on the Fp1 channel. The remaining noise-free epochs were distributed into four sERP waveform clusters associated with the four conditions mentioned earlier. Each sERP cluster contained more than 250 individual epochs after artifact rejection (300 before epoch rejection). The average number of rejected epochs over subjects was 33 ± 11. After the noisy epochs’ rejection, the number of remaining epochs could be unbalanced across the four clusters. To address this, we rejected the last epochs of larger clusters to form a database with an equal number of averaged sERP waveforms per cluster. Averaged sERPs were down-sampled by a factor of 8, resulting in 90 samples for the post-stimulus interval. These values were used as features in the subsequent steps to form the feature vectors for classification. Therefore, the features used for tactile attention classification in our system were the amplitudes of sERP extracted for each stimulated location while one of them was attended (and the other was ignored). Since a single sERP waveform comprises 600 ms of post-stimulus window, i.e., 90 samples of sERP amplitudes per location, but not all samples are relevant for attention task classification, to select relevant features, we employed a feature screening method based on statistical analysis of data collected in the training blocks.

### 2.5. Feature Selection Algorithm

The feature vectors used for classification in our system comprised sERP amplitude samples extracted from both locations, attended and ignored, during a single attention task. The feature screening method was employed to select the most relevant sERP signal samples that contribute the most to the classification process and that differ the most depending on the attention focus on one of the stimulated locations.

For feature selection, we employed a method of screening sERP waveforms collected during the BCI training phase for statistical differences using the Wilcoxon rank-sum test. The goal was to compare 10 epochs’ average sERP signals at each time instant (time-series index) between the clusters: ADSD vs. AVSD conditions and AVSV vs. ADSV conditions. The screening process aimed to reduce the number of samples in the feature vectors, identifying sERP components sensitive to the attention task for each stimulation hotspot independently.

This screening process identified time instants (time-series index) associated with statistically significant sERP amplitude changes induced by the tactile attention task (statistical significance threshold: *p* < 0.05). This resulted in a different set of time-series indices for each stimulation location (indices D—iD; and indices V—iV) for each channel of a single subject.

**Figure 3:**
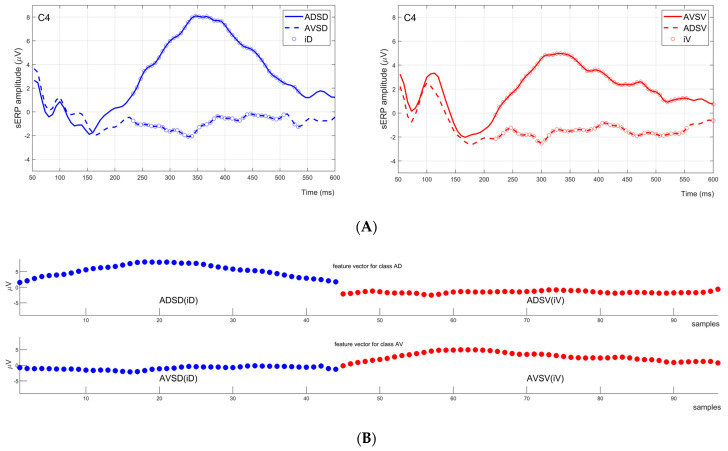
Feature vector generation example for one channel (C4) of one subject. (**A**) Two graphs in the first row of the figure are sERP responses for ADSD and AVSD conditions (**left**) in blue, and AVSV and ADSV conditions (**right**) in red. Indices denoting statistically significant sERP amplitude changes induced by tactile attention task are marked with circles: iD with blue circles on the left graph and iV with red circles on the right graph. (**B**) The bottom two plots of the figure represent feature vectors for classes AD and AV. The feature vectors for classes AD and AV were formed by creating a flattened array of [ADSD(iD),ADSV(iV)] and [AVSD(iD),AVSV(iV)], respectively.

Amplitude values at the selected subset of indices were extracted for each condition (ADSD(iD), ADSV(iV), AVSD(iD) and AVSV(iV)), and for each EEG channel. Feature vectors for two classes (target D—class label AD; and target V—class label AV) were formed by creating a flattened array of sERP amplitude values by joining the pairs [ADSD(iD), ADSV(iV)] for class label AD and [AVSD(iD),AVSV(iV)] for class label AV, respectively. The visualized example of the feature extraction (index identification) and feature vector formation is visually presented in Figure 3.

### 2.6. BCI Classifier Training

A separate support vector machine (SVM) classifier for each of the 5 EEG channels was trained with feature vector data. The SVM classifier employed a radial basis kernel function, and the kernel scale was optimized using a heuristic procedure. During classifier training, a leave-one-out cross-validation procedure was implemented to estimate BCI performance for each channel based solely on the training data. The EEG channel with the highest estimated accuracy was selected as the main feedback source for the subject during the online test, while the classification results of all 5 EEG channels from sensorimotor areas were displayed to the experimenter.

### 2.7. Online BCI Tests

Following the BCI classifier training, the online “BCI test phase” was initiated. During the online tests, the experimenter determined the order of target locations for each trial, which was pseudo-randomized. The same EEG preprocessing steps described earlier were applied online. Consecutive single epochs were processed online until 10 noise-free epochs were collected per stimulation location. The trial rejection criteria remained consistent with those applied during the BCI training phase. Averages of 10 epochs per location were formed, and amplitude values at indices iD and iV, identified during the training phase for each EEG channel, were selected from the 10 epochs’ average sERP for D and V locations, respectively. EEG channel feature vectors were formed and classified online. The classification results for each EEG channel were displayed to the experimenter, while the patient observed the classification result (AD or AV) of the single channel identified during classifier training, as explained in the previous section.

### 2.8. Data Analysis

During the online test, the BCI system provided a classification output for each EEG channel after collecting 20 epochs (10 epochs of attended D stimuli while unattended V stimuli, or vice versa). Consequently, each trial during the BCI test phase involved a binary classification problem of a feature vector formed from flattened 10 epochs’ average sERP amplitude values at predetermined indices from both stimulation hotspots during a single attention task: attended D or attended V location. Therefore, online BCI performance was assessed by classification accuracy for each EEG channel—Acc(ch), calculated as follows:

(1) $$Acc\left(ch\right)=\frac{TP\left(AD\right)+TP\left(AV\right)}{TP\left(AD\right)+TP\left(AV\right)+FP\left(AD\right)+FP\left(AV\right)}$$

where TP(AD) is the number of correctly classified trials of attended D location; TP(AV) is the number of correctly classified trials of attended V location; FP(AD) is the number of trials of attended D location misclassified as attended V; and FP(AV) is the number of trials of attended V location misclassified as attended D.

Descriptive statistics were performed on all variables. Continuous variables were presented as mean and standard deviation (SD) or median and an interquartile range (Q1–Q3), where Q1 and Q3 are the first and third quartiles. The Kolmogorov–Smirnov test was applied to test the normality of variables. Depending on the normality, a *t*-test for independent samples, *t*-test for paired data or Wilcoxon’s signed-rank test were used to compare continuous variables. To analyze the relationship between variables, the Pearson and Spearman correlation coefficients were used. The Pearson correlation coefficient was used for testing the linear relation between variables, while the Spearman correlation coefficient was used for testing the monotonic relation between variables. The point-biserial correlation coefficient was used to test the strength of association between a continuous variable and a binary variable. The threshold of significance in all tests was set at 5%.

## 3. Results

**Figure 4:**
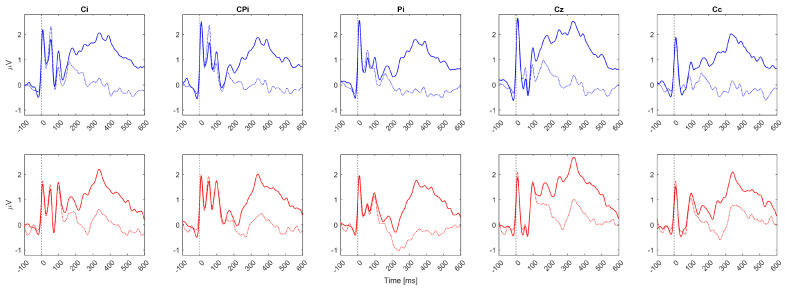
Grand average sERP waveforms over all patients for 5 EEG channels. Zero on the time axes marks the stimulus onset (vertical black dotted lines). The solid lines (solid blue or red) represent the attended condition, while the colored dashed lines (dashed blue or red) represent the unattended condition. The top row of subplots represents sERP responses associated with mixed radial nerve stimulation when the stimuli were attended (solid blue lines) vs. unattended (dashed blue lines). The bottom row of subplots represents sERP responses associated with mixed median nerve stimulation when the stimuli were attended (solid red lines) vs. unattended (dashed red lines). EEG channel labels are presented in the subplot titles. Ci and Cc represent ipsilasional and contralesional channels out of C3 and C4, i.e., if the patient had right body side paresis, Ci was C3, and Cc was C4; meanwhile, if the patient had left body side paresis, Ci was C4, and Cc was C4. The same rule was applied for labels CPi (CP5/CP6) and Pi (P3/P4).

For validation of neurophysiological nature of the classified responses, here, we present the morphology of grand average sERP responses over all patients and noise-free epochs recorded in this experiment. The grand averages are created for each condition and EEG channel separately and are displayed in Figure 4.

Since subjects with left UL impairment had different EEG channel configuration compared to subjects with right UL paresis, the following channel labels were applied for generalizing the results: Ci, CPi, Pi, Cz, Cc, where Ci was the ipsilesional channel out of C3 and C4; CPi was the ipsilesional channel out of CP5 and CP6; Pi was the ipsilesional channel out of P3 and P4; and Cc was the contralesional channel out of C3 and C4. Based on the grand average sERP waveform morphology (Figure 4), overlapping early sERP components between the conditions and the start of a mismatch between the attended and unattended conditions were observed after around 150 ms over all channels and both stimuli locations. Also, the amplitude increases in sERP components after 150 ms in the attended condition are in line with our previous results in healthy participants, which exhibited a similar activity pattern [34].

The results of online BCI tests showing the online BCI performance accuracy for all subjects and EEG channels are summarized in Table 2. The results of online BCI accuracy reveal that maximum classification accuracy was obtained in nine subjects from the channels covering the ipsilesional side. Only for the patient with ID 6 was maximum accuracy achieved for channel Cz, while none of the subjects obtained maximum accuracy for the contralesional channel. This was also reflected in the median classification accuracy per channel, where the lowest median value was obtained for channel Cc (Table 2). For further analysis, the channels were grouped into two subsets (subset 1: Ci, CPi, Pi; and subset 2: Cz, Cc) for each patient. The maximum accuracy for each subset was extracted and statistically compared using Wilcoxon’s signed-rank test. A significant difference between the maximum accuracies of subsets was obtained (*p* < 0.05), while the median (Q1–Q3) for subset 1 was 82.5% (75–90), and for subset 2, it was 72.5% (65–85). The result of offline analysis further confirmed that ipsilesional channels significantly outperformed central and contralesional channels in the accuracy of BCI control.

When patients were grouped by gender, the statistical analysis revealed no significant difference (*p* > 0.05) between the groups in terms of age, clinical assessment results, impaired side or BCI accuracy per single channel or maximum BCI accuracy. Similarly, when patients were grouped by impaired side, there was no statistical difference (*p* > 0.05) between the groups for either of the parameters. Correlation analysis was conducted to assess the potential relations between clinical assessment data (Table 1) and the maximum achieved online BCI accuracy per patient over EEG channels (Table 2). None of the correlation tests reached a significant correlation (*p* < 0.05); however, we will report the correlation coefficients as an indication of the type of relation between the variables. The positive correlation coefficient value (0.23) was obtained between maximum accuracy and FM-m, meaning that subjects with higher FM-m achieved higher maximum accuracy. Likewise, positive correlation coefficient values were obtained between maximum accuracy and FM-s (0.20), ARAT (0.20) and Barthel index (0.33). A negative correlation between maximum accuracy and two-point discrimination test results was detected (−0.31), indicating that patients with discrimination results <6 mm achieved higher maximum accuracy than patients with >10 mm discrimination results. A positive correlation between maximum accuracy and stereognosis assessment results was detected (0.36), meaning that patients with intact stereognosis results achieved higher maximum accuracy than patients with astereognosis.

## 4. Discussion

Given the importance of proprioception for motor control, it has been argued that therapies aiming to restore motor function after injury should also focus on training the proprioceptive sense [4]. The motivation for this work was the deficiency of somatosensory training guidelines and the fact that the employed methods lack a sound theoretical or empirical basis, which may have contributed to the lack of attention to sensory rehabilitation [35]. Therefore, this work is a step toward introducing novel quantitative and evidence-based therapeutic methods in the domain of somatosensory interventions. More specifically, our BCI enables a direct online assessment of the selective attention task success, both to the patient and the therapist during the therapeutic intervention. In this manner, the system provides a unique opportunity to directly assess whether the patient is performing the selective attention task in the expected manner during therapy. Moreover, by providing feedback to the user, there is a possibility of instructing the user to modify the attention strategy to increase performance.

In the context of BCI research, very limited number of previous systems for stroke rehabilitation employed mental task execution in parallel with somatosensory stimulation for BCI control [36,37,38]. In such studies, the aim of introducing somatosensory stimulation was mainly to improve the performance of motor imagery task. Therefore, previous BCI studies did not specifically recognize or consider the possible therapeutic effects of somatosensory stimulation and mental tasks of selectively focusing attention on the provided stimuli as a tool to facilitate perceptual learning and modulate sensory cortex plasticity, which is the main conceptual contribution of the current study. The empirical contribution of this work is the first feasibility test of a novel BCI control concept in a target population of subacute stroke patients. The main methodological novelty of our work is a clinic-ready protocol and BCI intervention pipeline, comprising classifier training and BCI intervention phase with online feedback on attention task classification.

We demonstrated that patients completely naïve to the BCI system and mental tasks were able to follow instructions and achieve a maximum median (Q1–Q3) online classification accuracy of 85% (75–90) with a single EEG channel for BCI control (the best-selected channel out of five candidates for control). Statistical analysis of the maximum BCI accuracy over all recorded channels per patient revealed that channels placed over the ipsilesional hemisphere were significantly more likely to achieve maximum accuracy compared to Cz channels and the contralesional channel. This result aligns with the expected electrophysiological activation patterns, as the attention task was associated with the stimulation of the paretic limb, resulting in maximum accuracies generally achieved by ipsilesional cortex activation. The correlation analysis between the clinical assessment scores and maximum channel accuracy did not result in significant correlation; however, all observed trends (positive or negative correlation coefficients) were in line with our initial hypotheses. Specifically, positive correlations were obtained between maximum accuracy and FM-m score, FM-s score, ARAT score and Barthel index. Although the correlation results are not significant, and the values of correlation coefficients indicate weak correlation, the type of relation is consistently positive over all clinical scores, indicating that higher scores (i.e., lower impairment) are related to higher BCI accuracy. This is also evident in the observed negative correlation between accuracy and two-point discrimination test results, showing that patients with discrimination results <6 mm achieved higher maximum accuracy than patients with >10 mm discrimination results. Additionally, patients with intact stereognosis results achieved higher maximum accuracy than patients with astereognosis. This demonstrated that stroke-related impairments did not have a significant effect on BCI performance, affirming the applicability of the system.

In our earlier study [33], we tested the offline classification of sERP in healthy subjects. It is important to emphasize that unlike our previous tests with healthy subjects, the current study employed completely online methods and introduced a comprehensive pipeline for BCI intervention. Our previous paper assessed BCI performance in relation to various feature extraction and classification methods in an offline manner, using cross-validation procedures. However, offline methods serve only as an indicator of potential online BCI performance. Several factors, such as the translation from offline to online signal filtering, signal processing delays, online artifact identification and fatigue or habituation over time, may influence online BCI control and result in decreases in performance in comparison to offline methods. These aspects are explored for the first time in the current paper, demonstrating that translation to full online control is feasible, with performance comparable to previously reported offline results. The current findings, obtained by employing similar methods in a realistic clinical setting with stroke survivors in the subacute stage of recovery, and in an online BCI scenario, are comparable and even exceed the previous results observed in healthy participants for single-channel BCI control.

The slightly higher accuracies in the current study may arise from the fact that previous results in healthy subjects used a 400 ms post-stimulus interval for feature extraction, while in this study, it was extended to 600 ms, providing more information for classification. The decision to expand the trial window from 400 ms to 600 ms was based on an exploratory study of sERP responses induced by a selective endogenous tactile attention task in healthy participants, which revealed sERP mismatches in the attended vs. unattended condition exceeding the 400 ms window, even though the main components induced by the task, over all subjects, remained below 400 ms [34]. The purpose of the implemented screening procedure for feature selection was to identify the time indices at which sustained spatial attention significantly affected the sERP morphology (associated with the same electrical stimulation hotspot), as sERP morphology may vary between stimulation hotspots. The current results show similar sERP morphological patterns associated with the attention task compared to healthy individuals [34]. More specifically, the task-induced mismatch started, on average, around 150 ms after the stimulus onset, while larger sERP amplitudes were obtained for the attended condition, as visible in the grand average sERP waveforms presented in Figure 4.

The main limitation in the context of BCI control lies in the lower information transfer rate (ITR) associated with the sERP as a BCI control signal compared to other ERP modalities. The ITR of our BCI design [39], calculated based on the number of targets (2), the number of commands (1) and the time in seconds per decision (14 s, corresponding to 700 ms x 20 epochs), is 4.29 bpm. However, it is essential to emphasize that, for the specific BCI application discussed in this paper, ITR and operational speed are not critical features. The current study explores the possibility of a BCI-supported therapeutic sensory intervention via neurofeedback. The trains of electrical stimuli possess rehabilitation potential, and coupling the afferent input with top-down attention resources may enhance the restorative effects of stimulation according to previous knowledge [17,21]. Moreover, providing feedback to the subject is solely for the purpose of reminding and encouraging them to sustain the attention task during the intervention. As the task itself requires a certain duration (sustained attention task), more frequent feedback instants are unnecessary, and increasing the number of feedback instances (and thus the ITR) is not crucial for this particular use-case scenario. The priority of our use-case scenario is that the online BCI should provide accurate feedback on the ongoing changes in brain activity induced by both stimulation and the executed tactile attention task to the patient and the therapist. For the current application purposes, in the accuracy vs. speed trade-off, we chose to prioritize the accuracy of the provided feedback (i.e., BCI performance/classification) over the frequency of the feedback instants. However, the obtained results are still comparable to those reported in a limited number of tactile ERP-based BCIs, with ITR values ranging from 2.9 bpm to 6.95 bpm and maximum accuracies ranging from 80% to 95%, as identified in the literature [40,41,42]. Moreover, all previous tactile BCI studies employed a multichannel design ranging from 4 to 14 EEG channels, while our study explored the possibility of a single-channel BCI control, which makes it more robust and applicable in daily clinical practice. Our results remain comparable to the state of the art in ITR and accuracy for tactile BCIs. This is noteworthy, given that we obtained these results using a single EEG channel and studied a patient population suffering from UL motor and sensory impairments, where a decrease in performance might have been anticipated. Furthermore, there are possibilities of improving the ITR and/or accuracy with multichannel features, which are open for future research. If the current BCI control methods are adapted for different applications, in which the speed of operation is a more important factor, such as sERP-based brain switches, the number of averages per trial can be reduced, which would increase the ITR. Moreover, calibration time may be further reduced using novel methods specifically designed for ERP-based BCIs [43].

The presented results demonstrate the feasibility of online sERP-based control in 10 stroke survivors, highlighting the easy deployment of the novel BCI sensory intervention pipeline within a clinical setting. Future work will involve testing the therapeutic effects of this BCI intervention.

## 5. Conclusions

This study presents the clinical feasibility trial of a novel BCI intervention designed to introduce a top-down approach into classical sensory training following stroke. The BCI system relies on repeated neuromuscular electrical stimulation, aiming for targeted closed-loop sensory cortex plasticity modulation and gating perceptual learning. The feasibility of an online single-channel BCI control was tested in ten stroke patients in the subacute phase of recovery, resulting in single-channel attention task classification accuracy ranging from 70% to 100% across patients. The study lays the groundwork for future research exploring the therapeutic effects of the BCI intervention, aiming at improving sensory training outcomes following stroke. The described methods introduce a novel BCI control paradigm and its clinical applicability, which may also have relevance in assistive BCIs for communication in severely disabled users. The main novelties relevant to the BCI field, as introduced by this study, include the new concept of top-down intervention for sensory training, a novel BCI control signal (sERP) tested for online control in subacute stroke patients, a new electrotactile BCI control paradigm utilizing a stream of equiprobable spatially distributed stimuli over the paretic forearm of the patient, mimicking standardized sensory discrimination training protocols, and a clinic-ready BCI pipeline for sensory training after stroke.

## Figures and Tables

**Table 1:** Demographic and clinical assessment data of the 10 stroke patients: ID, age (years), gender, CVI type, time since CVI (months), Fugl-Meyer motor score for upper extremity (FM-m), Fugl-Meyer sensory score for upper extremity (FM-s), ARAT, Barthel index (BI), Ashworth score for hand (AS), two-point discrimination distance (2-PD) and stereognosis assessment.

Patient ID	Age (y)	Gender	CVI Type	Time Since CVI (m)	FM-m	FM-s	ARAT	BI	AS	2-PD	Stereognosis
1	61	f	Ischemic	3	51	11	53	90	1	>10 mm	Astereognosis
2	67	m	Ischemic	5	55	12	52	95	1	<6 mm	Intact
3	61	f	Ischemic	1	38	10	29	90	1	<6 mm	Intact
4	65	m	Ischemic	2	62	12	57	100	1	<6 mm	intact
5	47	m	Ischemic	1	63	6	57	100	1	>10 mm	Astereognosis
6	63	m	Ischemic	5	29	10	15	90	1	>10 mm	Intact
7	46	f	Ischemic	2	58	12	57	95	1	<6 mm	Intact
8	46	f	Ischemic	5.5	63	12	57	95	1	<6 mm	Intact
9	69	m	Ischemic	4	64	12	57	95	1	<6 mm	Intact
10	60	f	Ischemic	1.5	48	11	48	70	1	<6 mm	Intact

**Table 2:** Summary of online BCI test results. Patient ID, impaired body side (left/right) and final electrotactile pulse amplitudes used for locations D and V are included in the first three columns. Columns 4–8 include online BCI accuracy per single EEG channel. Column 9 includes maximum BCI accuracy over all single channels for each patient. EEG channel labels including subscript “i” (Ci, CPi, Pi) cover the ipsilesional side (for right impaired body side: C3, CP5, P3; and for left impaired body side: C4, CP6, P4), and Cc channel label denotes contralesional side (for right impaired body side: C4; and for left impaired body side: C3). Columns 10 and 11 include maximum accuracies for channel subset 1, denoted as ch(i): Ci, CPi, Pi; and subset 2, denoted as ch(c,z): Cz, Cc, respectively.

Patient ID	Impaired Side	Amplitude D/V (mA)	Online BCI Accuracy (%)					Max. acc all ch (%)	Max. acc ch(i) (%)	Max. acc ch(c,z) (%)
			C_i_	CP_i_	P_i_	Cz	C_c_			
1	R	18/16	70	65	55	55	35	70	70	55
2	R	14/12	95	90	100	85	85	100	100	85
3	R	17/12	70	75	70	50	65	75	75	65
4	R	15/12	90	45	55	90	80	90	90	90
5	R	11/11	75	85	75	70	70	85	85	70
6	L	14/12	80	80	75	85	55	85	80	85
7	L	16/16	95	100	90	90	90	100	100	90
8	L	14/12	70	85	45	70	65	85	85	70
9	L	11/11	60	60	75	50	65	75	75	65
10	L	12/11	80	75	50	75	75	80	80	75
Median (Q1–Q3)			77.5 (70–90)	77.5 (65–85)	72.5 (55–75)	72.5 (55–85)	67.5 (65–80)	85 (75–90)	82.5 (75–90)	72.5 (65–85)

## Data Availability

The data supporting the findings of this study will be made available by the corresponding author upon reasonable request.
